# Workshops to Teach State-Tested Nursing Assistants About End-of-Life Care

**DOI:** 10.1093/geroni/igaa057.2230

**Published:** 2020-12-16

**Authors:** Kimberly Ogle

**Affiliations:** Miami University, Oxford, Ohio, United States

## Abstract

Given that almost 25 percent of U.S. deaths occur annually in long-term care facilities (U.S. Census Bureau, 2017), it’s imperative that frontline workers are given training and support they need to deliver good, person-centered care at the end of life. Inadequate end-of-life (EOL) care may lead to unrelieved suffering and undignified deaths (Bukki, Neuhaus, Paa, 2016). Furthermore, nursing staffs have knowledge gaps and low confidence regarding end-of-life care and they may underestimate its complexity (Pfister, Markett & Muller, 2013). With the growing population of older adults, improving end-of-life care in long- term care facilities needs to be a priority. This research explored the needs of State Tested Nursing Assistants (STNAs) working in long-term care and their knowledge regarding EOL care. Based on the findings of this research, workshops were developed to better educate the STNAs regarding care of the dying and to enhance the EOL care for long term residents.

